# De-novo assembly and comparative analysis of the complete mitogenome of traditional Chinese medicine *Strobilanthes sarcorrhiza*

**DOI:** 10.1186/s12870-025-06731-3

**Published:** 2025-05-21

**Authors:** Yujie Shi, Ping Li, Jian Sun, Meixin Li, Jingyong Jiang, Yue Xin, Zhen Chen, Wei Zeng

**Affiliations:** 1 Zhejiang Provincial Key Laboratory of Plant Evolutionary Ecology and Conservation, College of Life Sciences, Taizhou University, Taizhou, 318000 China; 2 Huangyan Forestry Technology Promotion Station, Taizhou, 318000 China; 3 Zhejiang Research Institute of Traditional Chinese Medicine, Hangzhou, 310023 China; 4 Institute of Horticulture, Taizhou Academy of Agricultural Sciences, Taizhou, 318000 China

**Keywords:** De-novo assembly, *Strobilanthes sarcorrhiza*, Mitochondrial genome, Molecular marker, Chinese medicine identification

## Abstract

**Background:**

*Strobilanthes sarcorrhiza* is a traditional medicinal plant known for its heat-clearing and kidney-nourishing properties. While its plastid genome has been reported, there is a scarcity of genetic information regarding its mitogenome, leading to unclear phylogenetic relationships. We sequenced and assembled the complete its mitogenome and conducted a series of genetic analyses in conjunction with the plastid genome to gain a better understanding of the species’ genetic background.

**Results:**

The mitogenome comprised a linear structure spanning 617,134 bp. It included 35 protein-coding genes (PCGs), 19 transfer RNAs (tRNAs), and 3 ribosomal RNAs (rRNAs) that have been annotated. Additionally, 122 simple sequence repeats (SSRs) and 25 tandem repeats were identified. A total of 1,482 pairs of dispersed repeats were detected, which account for 17.58% of the entire mitogenome. Furthermore, 37 migration fragments between the mitochondrial and plastid genomes were discovered, consisting of 5 complete PCGs, 7 tRNAs, and 1 rRNA. Based on the analysis of 38 mitogenomes and 46 plastid genomes, the evolutionary relationship and phylogenetic position of *S. sarcorrhiza* were elucidated.

**Conclusions:**

This study has, for the first time, provided insights into the mitochondrial genomic characteristics of *S. sarcorrhiza* and clarified its phylogenetic position. These findings offered significant insights for the future identification and classification of this genus, as well as for the genetic breeding of medicinal plants.

**Supplementary Information:**

The online version contains supplementary material available at 10.1186/s12870-025-06731-3.

## Background

The Acanthaceae is one of the largest families within the Lamiales order [1]. Statistics indicate that it comprises approximately 250 genera and over 4,000 species worldwide, primarily found in tropical regions [2, 3]. The family exhibits a high degree of diversity in survival forms, encompassing xerophytes, hygrophytes, herbs, shrubs, vines, and even small trees, and plays a significant role in the flora of many areas [4]. Acanthaceae plants are prized for their ornamental value and are frequently utilized as horticultural flowers, examples being *Thunbergia grandiflora*, *Barleria cristata*, and *Cyrtanthera carnea*. Additionally, medicinal properties are a prominent feature of Acanthaceae plants; for instance, *Strobilanthes cusia* and *Andrographis paniculata* are known for their heat-clearing, detoxifying, blood-activating, and anti-swelling effects [5, 6]. Consequently, members of the Acanthaceae family are plants of significant ecological, medicinal, and ornamental importance, with vast potential for development and utilization. However, due to the extensive number of genera and species, widespread distribution, and unique lifestyles, the Acanthaceae has long been regarded as a challenging group to classify [7, 8]. As a result, there is no comprehensive monograph on the family available worldwide. Moreover, the varying nutritional and reproductive organs of these plants make it difficult to achieve a consensus on the classification system proposed by different scholars based on various morphological characteristics [4, 9], particularly for the two largest genera, *Strobilanthes* and *Justicia*, which have been subject to differing opinions both domestically and internationally [1, 7, 10].

The *Strobilanthes* genus is one of the largest within the Acanthaceae family, comprising approximately 400 species [7, 11]. China stands as a center of diversity for this genus, hosting around 130 species [12, 13]. Many of these species possess significant medicinal properties. The *S. sarcorrhiza* is one of the representative species of the genus, with its substantial fleshy roots utilized in traditional Chinese medicine to nourish yin, clear heat, and address kidney deficiencies [14, 15]. It is a widely circulated remedy in southwestern Zhejiang and a distinctive plant of the region [14–16]. In recent times, the demand and price for it have escalated. Nonetheless, the taxonomy and evolutionary relationships within this genus remain ambiguous, resulting in the marketing of roots from other plants, such as *Pseudostellaria heterophylla*, *Lophatherum gracile*, and *Silene tatarinowii*, as *S. sarcorrhiza*. This practice has compromised the safety of clinical treatments and has impeded the development and utilization of this plant genus.

Mitochondria generate energy via aerobic respiration within eukaryotic cells [17]. They are involved not only in regulating key metabolic processes such as cell differentiation, apoptosis, growth, and division but are also closely associated with significant traits like stress tolerance, plant growth vitality, and cytoplasmic male sterility [18, 19]. Mitochondria are an essential tool for studying eukaryotic evolution, species identification, genetic diversity, and breeding [18, 20, 21]. The mitogenome is smaller than the nuclear genome, with coding genes that are highly conserved and non-coding regions that are highly variable [22, 23]. Currently, research on complete plant mitogenomes lags significantly behind that of complete plastid genomes. Although the NCBI database contains nearly 15,218 complete plastid genomes, there are only 1,102 complete plant mitogenomes [24]. Different plant mitogenomes exhibit significant structural and content variations, nucleotide substitution rates, and repetitive sequences, resulting in complex structural types such as circular, branched, and reticular structures within the mitogenomes [22, 25]. The genome length varies from 66 Kb for *Viscum scurruloideum* to 12 Mb for *Larix sibirica*, making the assembly of plant mitogenomes challenging [26, 27]. Consequently, most plant systematics research concentrates on nuclear and plastid genomes, and the complete assembly of plant mitogenomes remains a bottleneck in evolutionary biology. With the advancement of high-throughput sequencing technology and the emergence of the next generation of systematic genomics, numerous software programs suitable for mitochondrial genome sequencing and assembly have been developed, such as GetOrganelle [28], GSAT [29] and PMAT [30]. These advancements have made mitogenome sequencing and assembly more accurate and efficient, providing robust technical support for a deeper understanding of the genetic characteristics and phylogeny of species.

Only two mitogenomes (*Avicennia marina* and *Echinacanthus longipes*) have been reported in the Acanthaceae family [31]. However, to date, the mitogenome of the genus *Strobilanthes* has not been documented, and this gap in knowledge has significantly impeded phylogenetic research and the utilization of molecular resources for this genus. Consequently, there is an urgent need to analyze the mitogenome of *S. sarcorrhiza* to further acquire information that will aid in future genetic evolution, phylogeny, and conservation strategies.

We assembled and annotated the mitogenome of *S. sarcorrhiza*, and analyzed its genetic characteristics, repetitive sequences, codon usage bias, RNA editing sites, non-synonymous substitutions (Ka)/synonymous substitutions (Ks) ratios, and sequence transfers. Multicollinearity analysis was used to compare it with six related species to determine sequence rearrangements during evolution. Furthermore, based on 38 mitogenomes and 46 plastid genomes respectively, the evolutionary relationship and genetic background of the family Acanthaceae were further clarified. The purpose of this study is to analyze the mitogenome and provide insights for future research on genetic variation, phylogeny, and breeding of this species. Ultimately, it will also lay the groundwork for the protection and utilization of medicinal economic plants.

## Methods

### Plant sample collection and sequencing

The leaves of *S. sarcorrhiza* were collected from the planting greenhouse of Taizhou University (120°23.37’ N, 28°39.46’ E) in Taizhou City, Zhejiang Province, China, and stored in a -80℃ refrigerator for subsequent use. We utilized a modified CTAB method to extract total genomic DNA from the plants [32]. The DNA concentration was measured using a Nanodrop and a Qubit Fluorometer, and its purity and integrity were tested with 1% agarose gel electrophoresis. Subsequently, high-quality genomic DNA was sequenced using the Illumina NovaSeq X plus platform for second-generation sequencing and the Pacific Biosciences Revio platform for third-generation sequencing. In the end, we obtained WGS short reads and HiFi long reads, respectively.

### Assembly and annotation of mitogenome

Utilizing HiFi sequencing data, we assembled the mitogenome of *S. sarcorrhiza* using PMAT v1.5.3 [30]. The parameters and pattern employed by PMAT were “-st hifi -g 820m -m” and the “autoMito” model, respectively. The frequency of 19-kmers was generated from clean WGS reads using the jellyfish v2.2.7 tool [33]. Subsequently, the GenomeScope software [34] was utilized to evaluate the genome’s characteristics. Furthermore, we inferred the ploidy of the its genome based on k-mer data using the Smudgeplot v0.4.0 software [35].

We utilized the Bandage v0.8.1 tool [36] to visualize the original assembly graph and removed contigs corresponding to the nuclear and chloroplast genomes based on depth. Subsequently, we untangled the original assembly graph to obtain the *S. sarcorrhiza* mitogenome. The mitogenome was annotated using the online programs PMGA and GeSeq [37, 38]. We uploaded the assembled sequences to the PMGA online website (http://47.96.249.172:16084/annotate.html) in FASTA format and selected 319 plant mitogenomes as reference sequences for annotations. Simultaneously, the assembly data was uploaded to the Geseq online website (https://chlorobox.mpimp-golm.mpg.de/geseq.html), where the mitogenomes of Lamiales species were used as reference sequences to annotate the *S. sarcorrhiza* mitogenome, and other parameters were set to their default values. Additionally, tRNA and rRNA were annotated using tRNAscan-SE v2.0 and BLASTN [39, 40], respectively. All annotations were manually reviewed and corrected using Geneious v11.0.18 software [41]. Finally, the online tool PMGmap [42] was used to visualize the complete mitogenome with default parameters, including *cis-* and *trans*-splicing gene maps.

### Identification and analysis of sequence repeats

The SSRs in the mitogenome were identified using the online program MISA [43]. The minimum repeat numbers for mononucleotide (Mono), dinucleotides (Di), trinucleotides (Tri), tetranucleotides (Tetra), pentanucleotides (Penta) and hexanucleotides (Hexa) were set to 10, 5, 4, 3, 3, and 3, respectively. Dispersed repeats were detected through the online tool REPuter [44], which included four types: forward (F) repeat, reverse (R) repeat, palindrome (P) repeat and complementary (C) repeat. The Hamming distance was set to 3, and the maximum number of repeats and minimum repetition size were set to 5,000 and 30, respectively. Additionally, tandem repeats (TR) in the mitogenome were detected using the online program Tandem Repeats Finder with default parameters [45]. Finally, all repetitive components were manually inspected and corrected using the “Advanced Circos” module in Tbtools v2.142 software [46].

### RNA editing event detection

All PCGs in the mitogenome of *S. sarcorrhiza* were extracted. The editing events of C-to-U RNA from the PCGs were predicted through Deepred-Mt software [47], which employs a convolutional neural network (CNN) model. Finally, results with a threshold greater than 0.9 were considered reliable editing sites.

### Analysis of codon usage bias

The PCGs of eight mitogenomes (*S. sarcorrhiza*, *A. marina*, *Salvia miltiorrhiza*, *Osmanthus fragrans*, *Rehmannia glutinosa*, *Utricularia reniformis*, *Plantago ovata*, and *Boea hygrometrica*) from Lamiales were extracted using PhyloSuite v1.2.3 [48]. The “relative synonymous codon usage (RSCU) analysis” module in the online tool library (http://cloud.genepioneer.com:9919) was used to compare the codon usage preferences of PCGs in the different mitogenome, and their RSCU values were calculated respectively.

### Identification of migration sequences between mitochondria and plastids

There are frequent migration events between the mitochondrial genome and the plastid genome in higher plants. To detect mitochondrial plastid DNA transfers (MTPTs) in *S. sarcorrhiza*, we utilized Getorganelle v1.7.7 software [28] to assemble the complete plastid genome (accession number PQ631129). Subsequently, BLASTN software was employed to identify the homologous sequences between the mitogenome and the plastid genome, with the e-value ≥ 1 × 10^− 5^. The MTPTs with matching rates ≥ 70% and the lengths ≥ 30 bp were chosen for further manual annotation analysis. Ultimately, the results of the MTPTs were visualized based on the “Advanced Circos” module in Tbtools v2.142 software [46].

### Collinearity analysis of mitogenome

To compare collinearity between the mitogenomes of *S. sarcorrhiza* and those of other related plants, we retrieved the mitogenomes of six Lamiales species (*A. marina* PP908999; *S. miltiorrhiza* NC_023209; *U. reniformis* NC_034982; *R. glutinosa* NC_086689; *E. longipes* PQ164709, PQ164710, PQ164711, PQ164712, PQ164713; *O. fragrans* NC_060346) from the NCBI database. Each mitogenome was compared in pairs with that of *S. sarcorrhiza* via MUMmer4 software [49], and only collinear fragments with an alignment length of at least 500 bp were kept for further analysis. The collinearity results were then visualized with NGenomeSyn v1.39 software [50].

### Phylogenetic analysis

To ascertain the phylogenetic position of *S. sarcorrhiza*, we constructed phylogenetic trees using both plastid and mitochondrial sequences, respectively. We retrieved the plastid genomes of an additional 46 species from the NCBI database. However, since the *Strobilanthes* genus lacks mitogenomes, we could only obtain sequences for 38 species (Table S1). We extracted 10 conserved mitochondrial PCGs and 20 conserved plastid PCGs using PhyloSuite v1.2.3 [48]. Subsequently, these shared PCGs were multiple aligned through MAFFT v7.149b software [51] and sequence trimming was performed using trimAl v1.2 software [52]. We used IQ-TREE v2.2.0.3 software [53] to build the maximum likelihood (ML) trees, with a bootstrapping value of 5,000 repeats. Based on the Bayesian Information Criterion (BIC) and Akaike Information Criterion (AIC) scores, the GTR + F + I + G4 model was selected as the optimal model for ML phylogenetic tree construction. Finally, the online platform ChiPlot [54] was used to visualize the phylogenetic tree of both plastid and mitochondrial genomes.

## Results

### General characteristics of the mitogenome

**Fig. 1:**
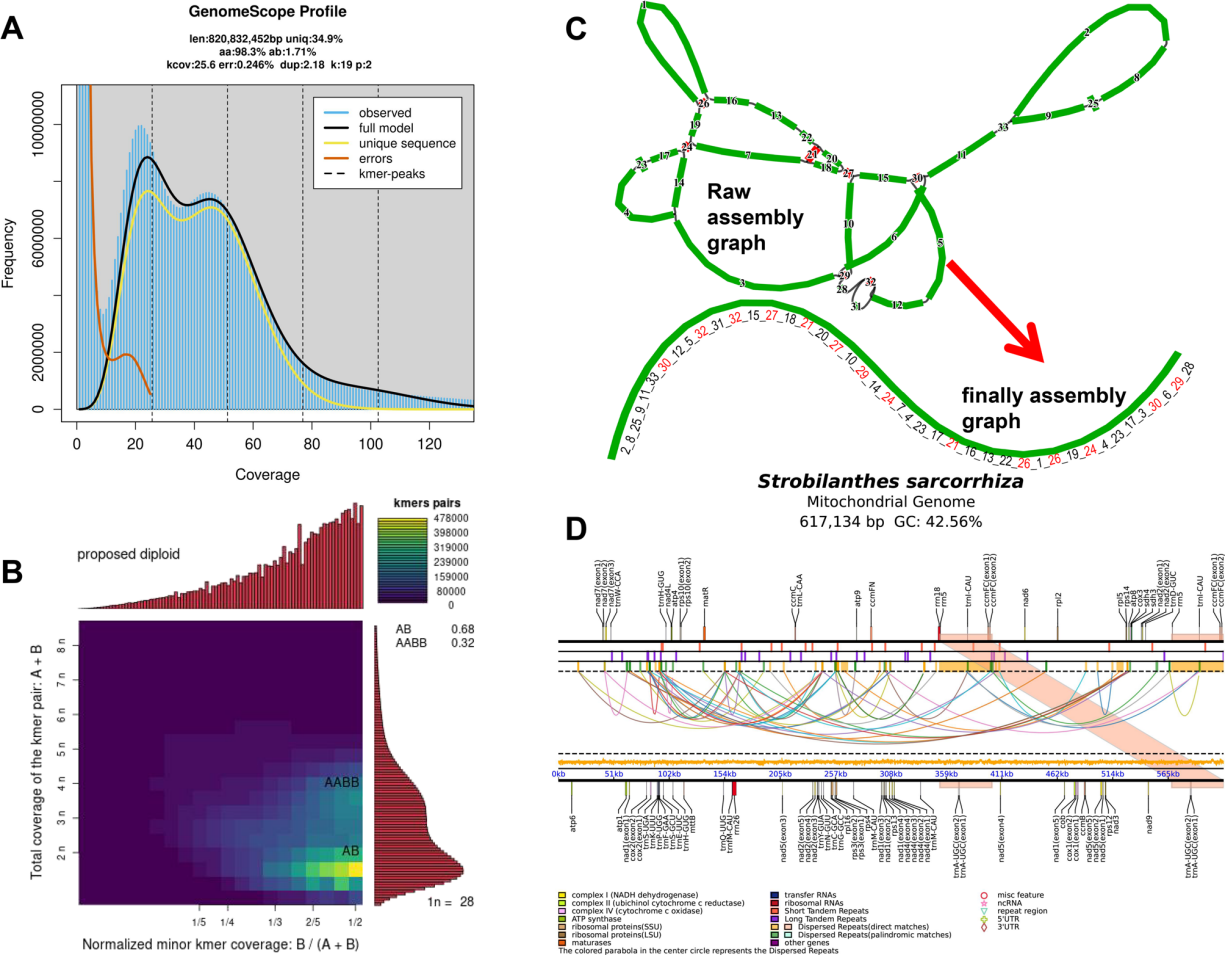
Assembly and annotation of the mitogenome of *S. sarcorrhiza*. (**A**) The genomic size was estimated based on k-mer analysis. (**B**) The genomic ploidy was inferred based on k-mer data. (**C**) The original and finally assembly graphs of the mitogenome. (**D**) The annotation of mitogenome

A total of 62.63 Gb (76.6×) Illumina clean reads, and 10.5 Gb (12.8×) of HiFi reads with an N50 of 18,337 bp, were generated (Table S2-S3). Initially, based on short clean reads, the genome size of *S. sarcorrhiza* was predicted to be 820 Mb and it might be a diploid species (Fig. 1A and B). Subsequently, a draft mitogenome was assembled using high-precision long-read sequence data. The repetitive sequences were sequentially unwrapped based on the coverage of contigs, resulting in a linear structure with a size of 617,134 bp (Fig. 1C). The GC content of the mitogenome was 42.56%, and the intergenic region accounted for 93.7% of the mitogenome, indicating a very sparse gene density (Fig. 1D). A total of 35 PCGs were annotated, including 24 core mitochondrial genes and 11 variable genes (Table S4). Additionally, 3 rRNAs and 19 tRNAs genes were annotated. The core genes comprised 5 ATP synthase genes, 4 cytochrome c biogenesis genes, 1 ubichinol cytochrome c reductase (*cob*), 3 cytochrome c oxidase genes, 1 maturase (*matR*), 1 transport membrance protein (*mttB*) and 9 NADH dehydrogenase genes. The variable genes included 3 large ribosomal proteins, 6 small ribosomal proteins, and 2 succinate dehydrogenase genes (*sdh3*,* sdh4*). Notably, the genes *ccmFC*, *rrn5*,* trnA-UGC*,* trnH-GUG*,* trnI-CAU* and *trnM-CAU* each had 2 copies. Among the 35 PCGs, 10 genes contained introns, with 5 genes (*ccmFC*,* cox1*,* cox2*,* rps10*,* rps3*) containing only 1 intron, and 5 genes (*nad1*, *nad2*, *nad4*, *nad5*, *nad7*) each containing multiple introns (Fig. 1D). Furthermore, additional analysis of the mitogenome revealed 7 cis-splicing genes within PCGs and 3 trans-splicing genes (*nad1*, *nad2*, *nad5*) within NADH dehydrogenase genes, indicating that these three genes may be related to adaptive evolution.

### Repeated sequence analysis of the mitogenomes between *S. sarcorrhiza* and related species

**Fig. 2:**
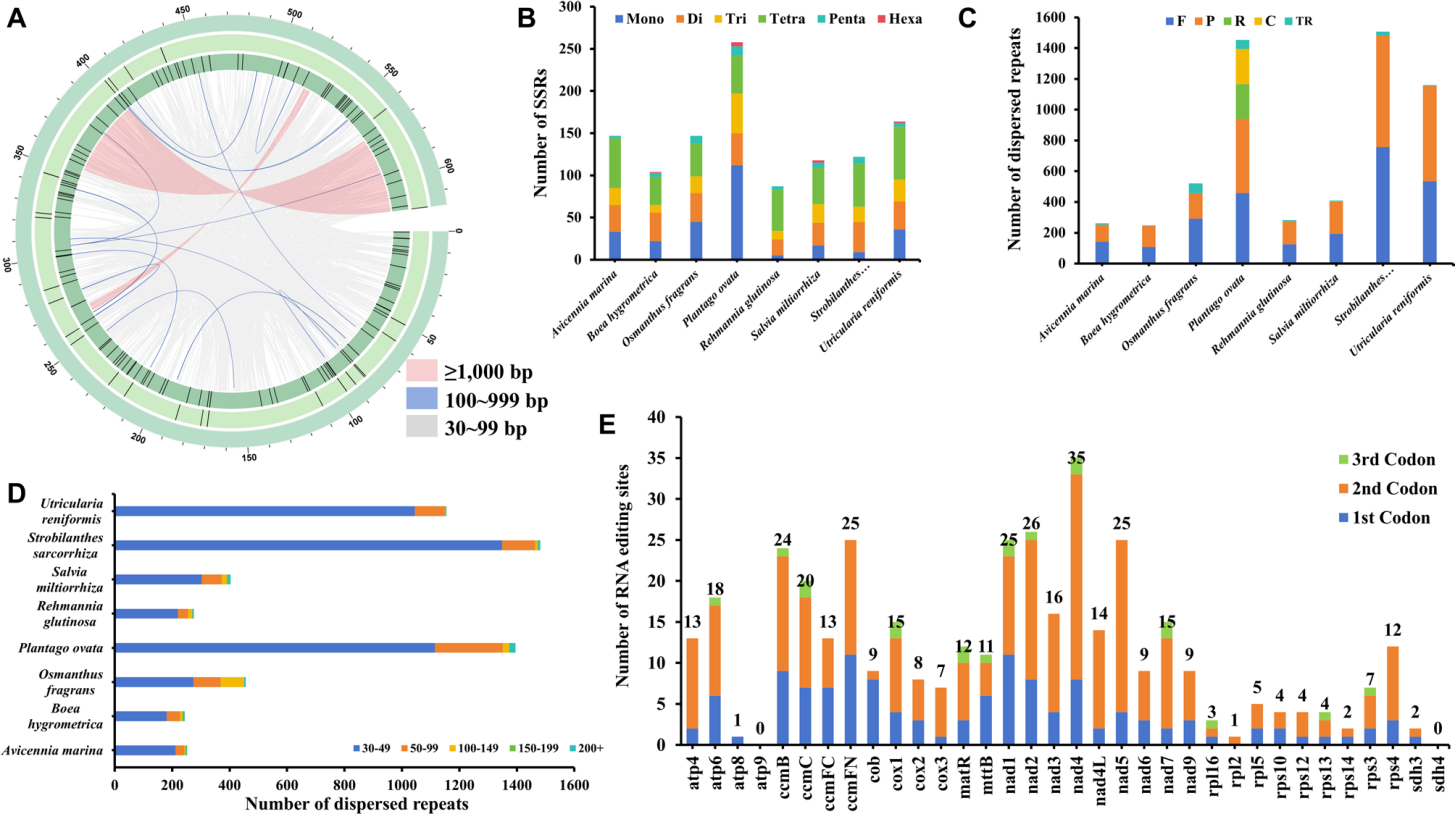
Identification of repetitive sequences and RNA editing sites in the mitogenomes. (**A**) Distribution of repetitive sequences in the mitogenome of *S. sarcorrhiza*. From the outside to the inside were the mitogenome, TRs, SSRs, and dispersed repeats. (**B**) Comparative analysis of SSRs loci among the mitogenomes of *S. sarcorrhiza* and 7 related species. (**C**-**D**) Comparative analysis of dispersed repeats loci among the mitogenomes of *S. sarcorrhiza* and 7 related species. (**E**) Identification of RNA editing events in the mitogenome

Repetitive sequences are prevalent in plant mitogenomes and are primarily categorized into three types: SSRs, dispersed repeats and TRs (Fig. 2A). The mitogenome of *S. sarcorrhiza* contained 122 SSRs, encompassing 5 types, with Tetra repeats being the predominant repeating unit (constituting 41.8% of all repeats). This is comparable to the mitogenomes of the other four Lamiales species (*A. marina*, *R. glutinosa*, *S. miltiorhiza*, *U. reniformis*) (Fig. 2B). In contrast, the other two related species (*O. fragrans*, *P.ovata*) predominantly feature Mono repeats, and *P. ovata* had a significantly higher total number of SSRs (258 loci) than the other seven related species (ranging from 87 to 164 loci).

In the mitogenome of *S. sarcorrhiza*, we identified 25 TRs, ranging from 5 in *B. hygrometrica* to 65 in *O. fragrans* (Fig. 2C). These TRs were short, with lengths varying between 25 bp and 74 bp, and were sparsely distributed. In contrast, dispersed repeats exhibited characteristics opposite to those of tandem repeats. They were evenly dispersed throughout the mitogenome and had longer lengths (Fig. 2A). The *S. sarcorrhiza* mitogenome contained 1,482 dispersed repeats, exceeding the number found in seven other similar species, ranging from 244 repeats in *B. hygrometrica* to 1,395 in *P. ovata*. The total length of these dispersed repeats was 108,514 bp, constituting 17.58% of the entire *S. sarcorrhiza* mitogenome. Most sequences were shorter than 50 bp (1,348 repeats, 90.96%), and only 7 repeats (0.47%) were larger than 200 bp, a pattern similar to that observed in the mitogenomes of seven other related species (Fig. 2D). Moreover, the types of dispersed duplication exhibited similar patterns among the eight related species. Except for *P. ovata* and *S. militorhiza*, which contained 4 types (F, R, P, C) and 3 types (F, R, P) respectively, all other species contained only F and P types. However, among the eight species, the F (accounting for 32.90-63.82% of all) and P (accounting for 34.55-55.74% of all) types still had the highest occurrence frequencies, a consistency observed across these species (Fig. 2C).

### Prediction of RNA editing sites in the mitogenome

We predicted potential C-to-U RNA editing sites for 35 PCGs in the *S. sarcorrhiza* mitogenome to gain a deeper understanding of gene expression (Fig. 2E). A total of 394 RNA editing sites were identified, with the *nad4* gene containing the most RNA editing sites (35 sites), followed by *nad2* gene (26 sites). Most ribosomal proteins had fewer editing sites, and the *rpl2* gene had just one editing site. Interestingly, the second codon was edited most frequently, with 248 sites (62.94% of the total), whereas the third codon had the fewest edits (19 sites, 62.94% of the total), significantly fewer than the first and second codons. Typically, these RNA editing events resulted in changes to the encoded amino acids, with most involving the conversion of hydrophobic amino acids, which can enhance protein stability.

### Analysis of selection pressure of shared PCGs from Lamiales species

**Fig. 3:**
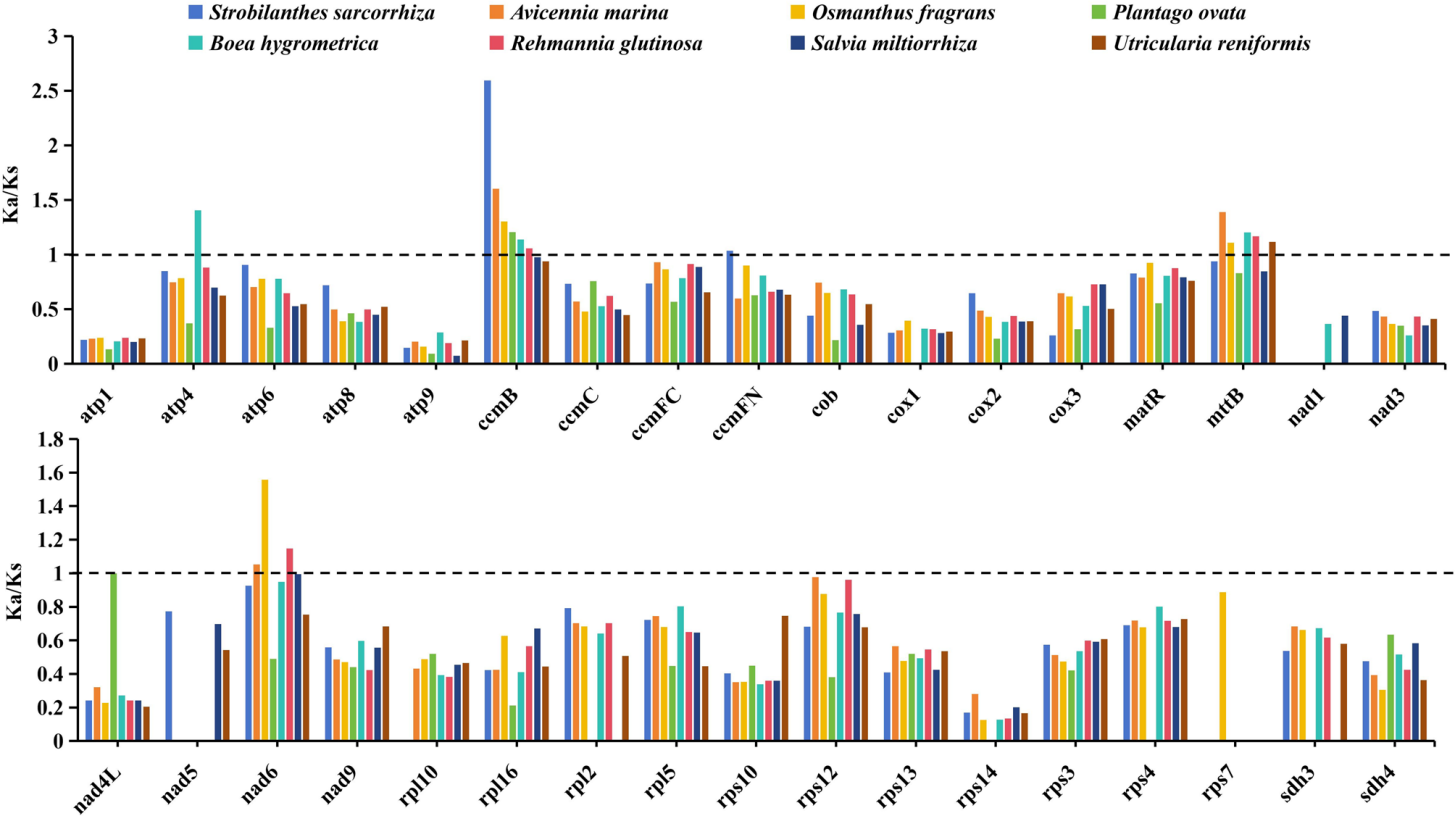
Selection pressure analysis of 34 PCGs from the mitogenomes of *S. sarcorrhiza* and 7 related species

To investigate the impact of selection pressure on the evolution of the mitogenome of *S. sarcorrhiza*, we selected seven closely related species and analyzed the ratio of Ka to Ks in the 34 PCGs shared with *S. sarcorrhiza* mitogenome (Fig. 3). The analysis of selection pressure revealed that the Ka/Ks ratio trend in the 34 shared PCGs was similar among the eight species, with most genes experiencing purifying selection, and the Ka/Ks ratio values were less than 1. Interestingly, only the Ka/Ks ratio values of three genes (*ccmB*, *mttB*, *nad6*) were higher than 1 in most species, and the *ccmB* gene in *S. sarcorrhiza* mitogenome exhibited the highest value (2.59), indicating that it had undergone potential positive selection in response to environmental adaptation.

### Analysis of codon usage bias

**Fig. 4:**
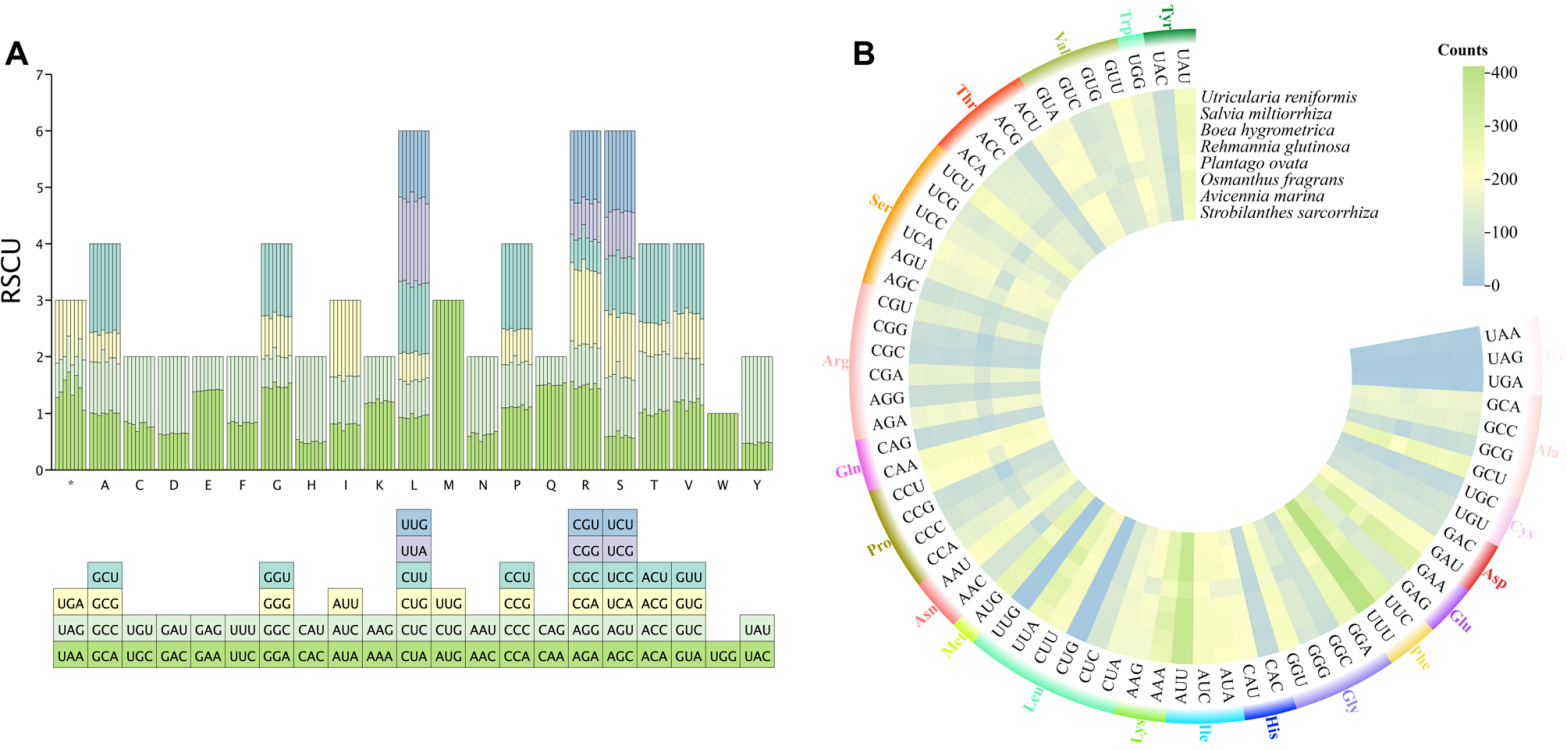
Codon usage bias analysis of mitogenomes from *S. sarcorrhiza* and 7 related species. (**A**) The RSCU values of *S. sarcorrhiza* and 7 related species. Each bar represents a species, from left to right, namely *S. sarcorrhiza*, *A. marina*, *O. fragrans*, *P. ovata*, *B*. *hygrometrica*, *R. glutinosa*, *S. miltiorrhiza*, and *U. reniformis*. (**B**) Statistics on the number of amino acids and codons corresponding to PCGs in different species

In total, 82,646 codons were identified from the mitochondrial PCGs of 8 Lamiales species, distributed in 8,817 in *P. ovata* and 10,829 in *A. marina*. There were 64 different types of these codons, encoding a total of 20 amino acids and 1 stop codon. Of these, UUU was the most commonly used codon, with 400–413 occurrences. Among the 20 amino acids, the trends of codon usages bias were consistent across the 8 species (Fig. 4B). Leucine exhibited the highest codon usage bias, with 1,071 to 1,152 codons, which accounted for 10.78% of the all. Serine followed, with 693-1,003 codons, representing 9.11% of the total, whereas cysteine had the fewest codons, with 144–168, constituting 1.48% of the total.

In addition, we calculated the RSCU values for the 64 codons across various species. The results indicated that 32 codons were utilized more frequently than anticipated, as evidenced by RSCU values exceeding 1 (Fig. 4A). Conversely, the remaining 32 codons were used less frequently than expected (RSCU < 1). Interestingly, both the lowest and highest preferences were observed in the same amino acid, methionine. Specifically, the codon AUG exhibited the most pronounced usage bias, whereas codons CUG and UUG demonstrated the weakest preference. Notably, tryptophan exhibited no codon preference (RSCU = 1). Overall, with the exception of tryptophan, all amino acids displayed codon usage preferences. Most amino acids possessed at least two distinct codons, with arginine, serine, and leucine each having six unique codons.

### Genes transfer within the organelle genome

**Fig. 5:**
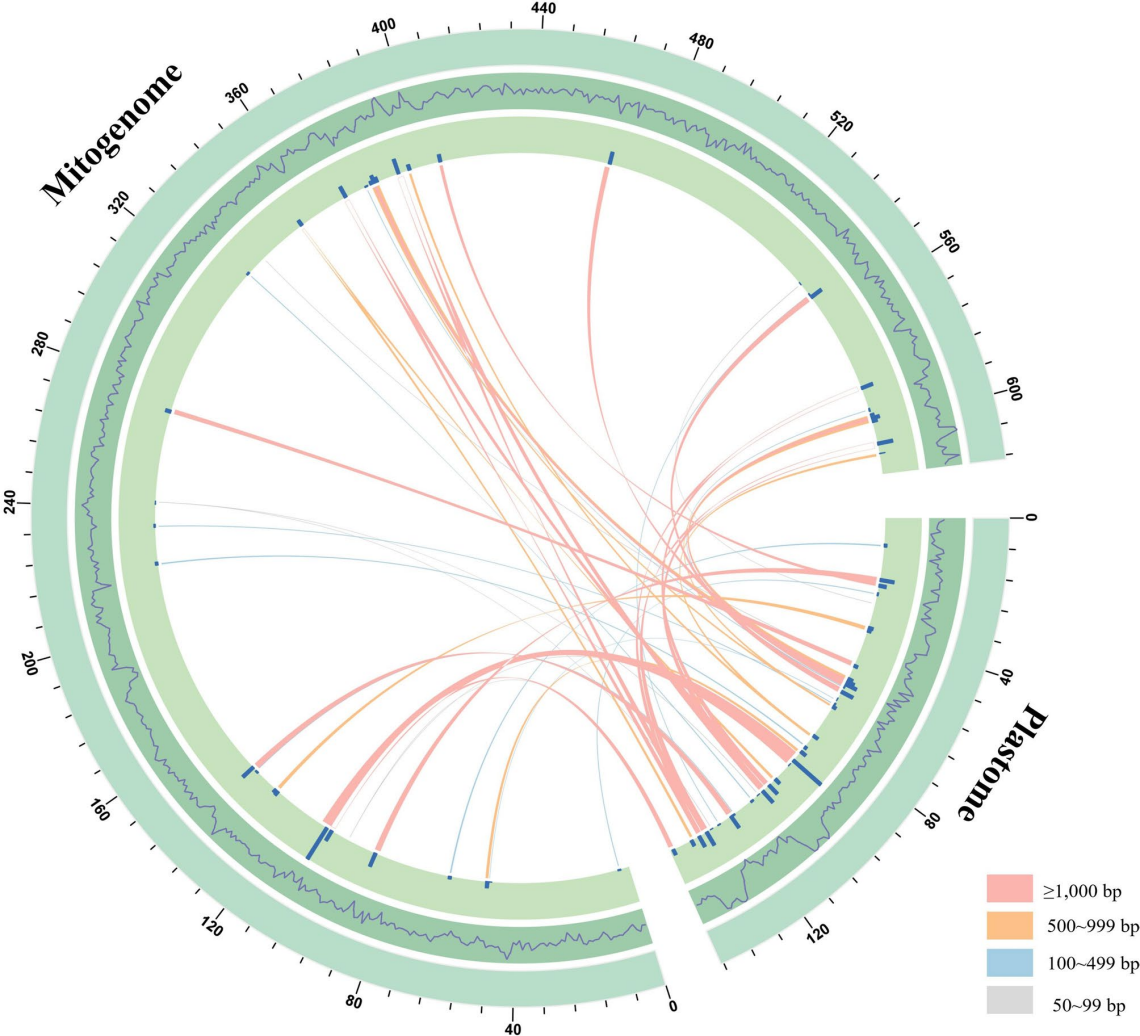
Detection of migration fragments between mitogenome and plastid genome in *S. sarcorrhiza*. Two arcs of different lengths represented the mitochondrial and plastid genomes. The curve on the outer circle represented the GC content of the two genomes, and the histogram on the inner circle represented the length of MTPTs. The line between the two arcs represents MTPTs. The detailed information was recorded in Table S4

The mitogenomes of higher plants contained a substantial number of sequences that have migrated from nuclear and plastid genomes. In the organelle genome of *S. sarcorrhiza*, we identified 37 MTPTs (total length of 34,978 bp), all with a similarity greater than 73%, accounting for 24.17% of the plastid genome and 5.67% of the mitogenome, respectively. Among these migration sequences, 21 fragments were larger than 500 bp, with the longest being 5,120 bp (Fig. 5). By annotating these fragments, a total of 34 plastid gene fragments were identified to be located in MTPTs (Table S4), including 22 PCGs fragments (*ycf1*, *ycf2*, *ycf4*, *psbA*, *psaA*, *psaB*, *psaI*, *petA*, *atpA*, *atpB*, *atpE*, *rpl16*, *rpl20*, *rpl23*, *rps18*, *rpoB*, *rpoC1*, *ndhA*, *ndhF*, *ndhH*, *accD*, *cemA*), 9 tRNA gene fragments (*trnA-UGC*, *trnD-GUC*, *trnH-GUG*, *trnI-CAU*, *trnI-GAU*, *trnM-CAU*, *trnN-GUU*, *trnW-CCA*, *trnV-GAC*), and 3 rRNA gene fragments (*rrn4.5 S*, *rrn16S*, *rrn23S*). However, only 13 genes (*psbA*, *psaI*, *ycf4*, *atpE*, *cemA*, *trnA-UGC*, *trnD-GUC*, *trnH-GUG*, *trnM-CAU*, *trnN-GUU*, *trnI-CAU*, *trnW-CCA*, *rrn4.5 S*) remained intact after transfer.

### Collinearity analysis of mitochondrial genomes

**Fig. 6:**
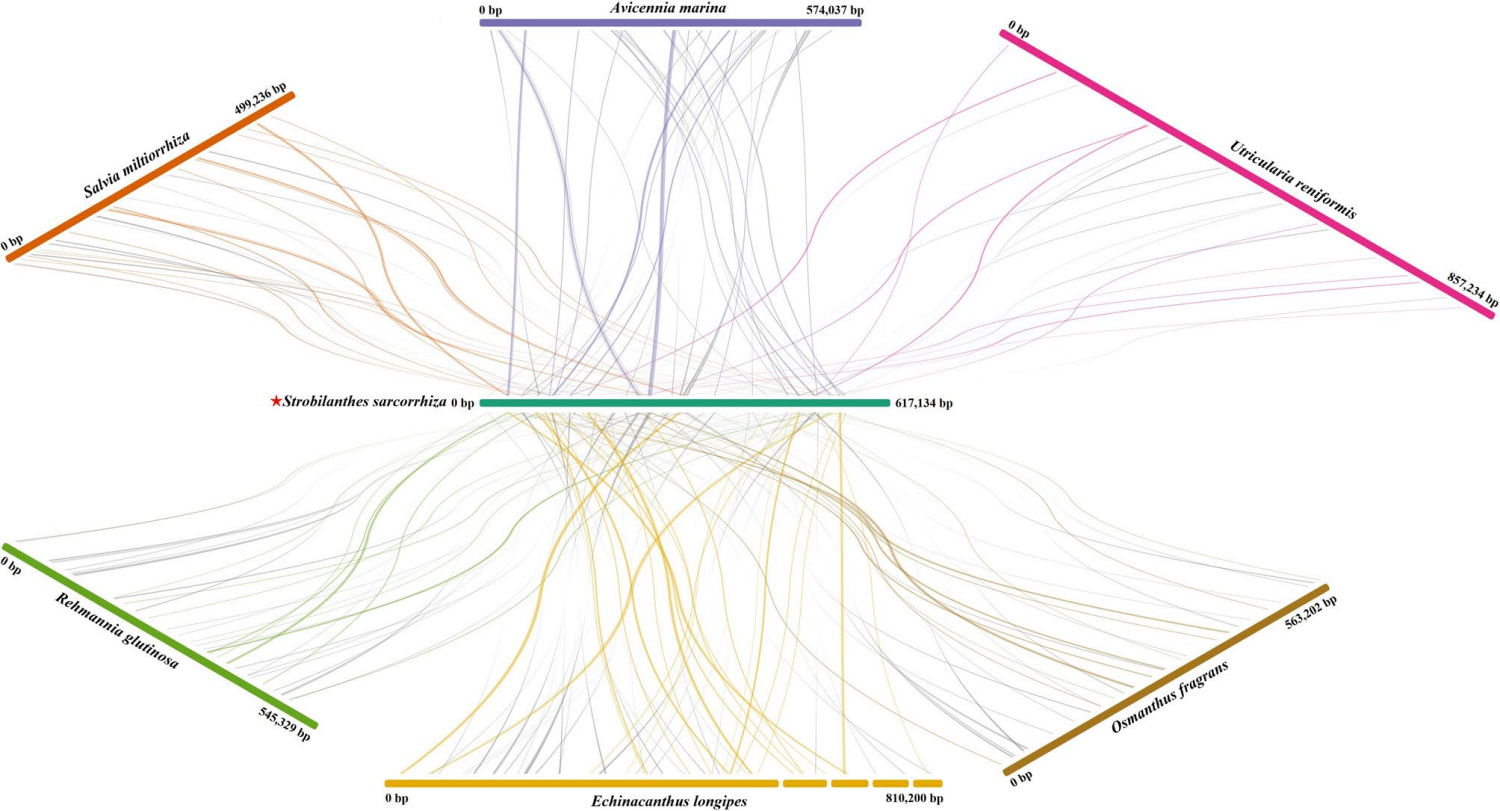
Collinearity analysis among the mitogenomes of *S. sarcorrhiza* and 6 related species. Different colored bars represented the mitogenomes of different plants. Among them, the colored line between the mitogenome of *S. sarcorrhiza* and other 6 species represented good similarity, and the gray line represented reversal. All colinear fragments were greater than 500 bp

In this study, we used the mitogenome of *S. sarcorrhiza* as the primary focus and conducted a collinearity analysis with the mitogenomes of six other related species (Fig. 6). Upon comparison with the mitogenome of *S. sarcorrhiza*, we identified 31, 43, 24, 38, 57, and 35 homologous fragments in *S. miltiorrhiza*, *A. marina*, *U. reniformis*, *R. glutinosa*, *E. longipes*, and *O. fragrans*, respectively, all of which were larger than 500 bp. Notably, the comparison between *E. longipes* and *S. sarcorrhiza* revealed the highest number of homologous fragments, with a total length of 90,241 bp, constituting 11.14% of the *E. longipes* mitogenome. Conversely, the collinearity between the *U. reniformis* and *S. sarcorrhiza* mitogenomes was the poorest, with only 24 homologous fragments (total length of 27,608 bp), representing 3.22% of the *U. reniformis* mitogenome. Furthermore, numerous sequence rearrangements were also identified within the mitogenomes of *S. sarcorrhiza* and the other species.

### Phylogenetic analysis

**Fig. 7:**
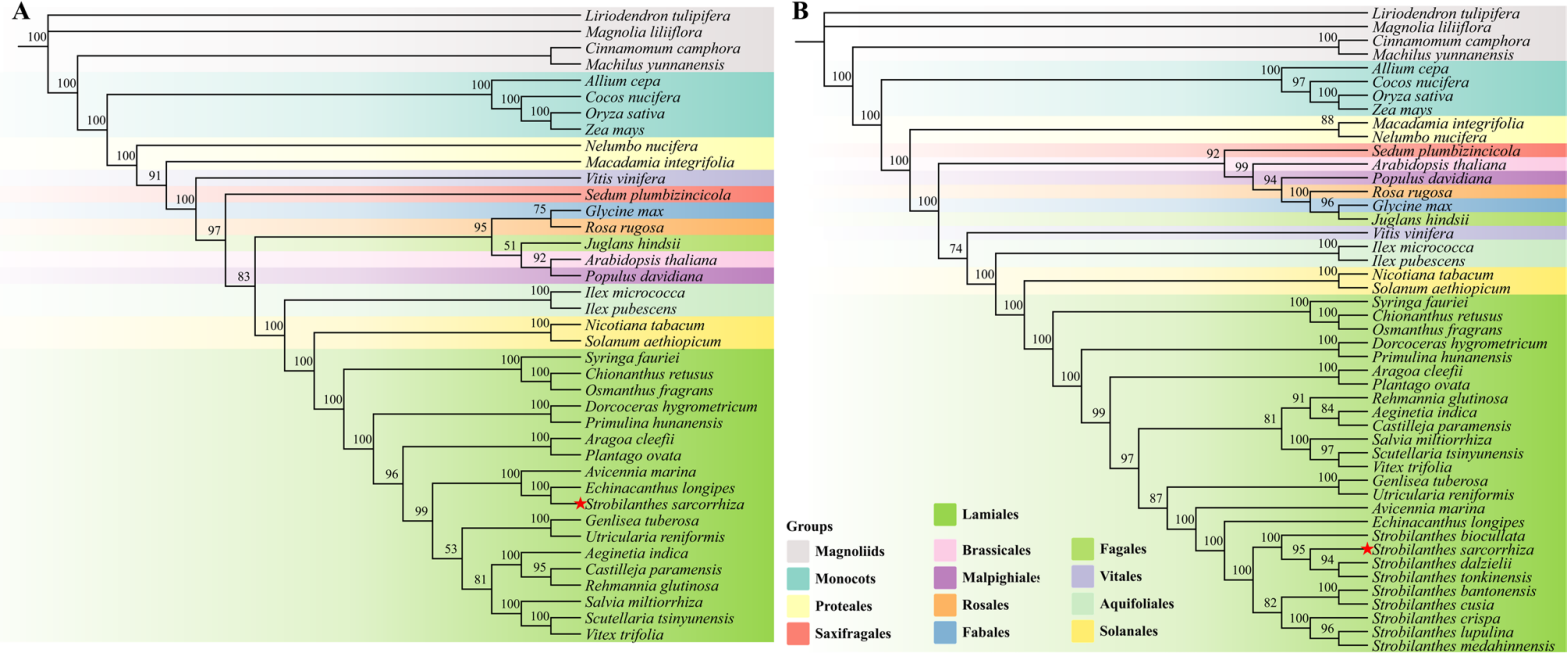
Phylogenetic analysis. (**A**) The ML phylogenetic tree constructed based on PCGs shared in the mitogenomes of *S. sarcorrhiza* and 38 species. (**B**) The ML phylogenetic tree constructed based on PCGs shared in the plastid genomes of *S. sarcorrhiza* and 46 species. Support rates for different nodes were displayed on branches

To further investigate the phylogenetic status of *S. sarcorrhiza*, an ML tree was constructed based on 10 conserved mitochondrial PCGs from 39 plant species (Fig. 7A). Phylogenetic analysis revealed that *S. sarcorrhiza*, *A. marina*, and *E. longipes* formed a clade (belonging to Acanthaceae), and together with other family species, constituted the Lamiales. The reconstructed ML tree proved that the phylogeny of *S. sarcorrhiza* was fully supported (bootstrap = 100), and the topology of the entire tree exhibited a high degree of consistency with the APG IV classification system. Due to the absence of mitogenomes from plants of the same genus as *S. sarcorrhiza*, we further examined the phylogenetic relationships within the genus using plastid sequences to reconstruct the phylogenetic tree. An ML phylogenetic tree was constructed based on 20 conserved plastid PCGs from 47 plant species (Fig. 7B). The results indicated that *S. sarcorrhiza* and 8 species from the same genus initially clustered into a branch, which then formed a clade with *A. marina* and *E. longipes*, and exhibited a closer genetic relationship with *S. tonkinensis*, *S. dalzielii*, and *S. biocullata*. The ML tree constructed from the mitogenomes and the plastid genomes showed good consistency in the phylogenetic relationship about *S. sarcorrhiza*, thus verifying the accuracy of the phylogenetic analysis.

## Discussion

Plant mitogenomes exhibit complex structural characteristics, with variations across different species, including linear, circular, branched and network structures [55]. In some species, multiple structures can coexist simultaneously due to high-frequency recombination mediated by repeated sequences [56, 57]. Numerous studies have elucidated the diversity of mitogenome conformations in various plants. For instance, the mitogenome of *Cinnamomum camphora* is a linear structure spanning 900,894 bp [58], whereas the *Populus simonii* mitogenome consists of three circular structures of different sizes [59]. The mitogenome of *Lilium tsingtauense* is particularly complex, spanning 1,125,108 bp and containing 27 independent circular chromosomes [60]. In this study, we successfully assembled the mitogenome of *S. sarcorrhiza* for the first time. Its linear structure diverges from those of other plants within the same family. For example, *A. marina* possesses a typical circular structure [31], and *E. longipes* features a multi-circular compound structure, suggesting that the mitogenomes of Acanthaceae plants may have undergone divergent evolution, contributing to the high plant diversity observed. The sizes of mitogenomes are influenced by the accumulation of various repetitive sequences and horizontal gene migration [61]. The mitogenome of *S. sarcorrhiza* spans 617,134 bp, which is comparable to that of *A. marina* (574,037 bp) and falls within the middle range of Lamiales mitogenome sizes (274,779 bp to 857,234 bp). Repeated and migrating sequences dictate the diversity in the sizes of Lamiales mitogenomes. In the *S. sarcorrhiza* mitogenome, 57 functional genes have been annotated, including 35 PCGs, 19 tRNAs and 3 rRNA genes. Notably, GC content is a crucial indicator for evaluating species evolution and impacts amino acids composition [62]. The GC content of the *S. sarcorrhiza* mitogenome is found to be approximately 42.56%, which aligns with the GC content range of Lamiales species (42.3-45.6%), suggesting that despite the structural and size diversity of Lamiales mitogenome throughout evolution, the GC content has remained relatively stable. Comparable outcomes are observed in Apiales species and Rosoideae species [55, 63]. In genetic research, the ratio of Ka to Ks for a gene can reflect its relative molecular evolution rate. Ka/Ks > 1, Ka/Ks = 1, and Ka/Ks < 1 indicate that the gene has been subjected to positive selection, neutral selection and purifying (negative) selection, respectively [64]. In our study, among the 34 PCGs from *S. sarcorrhiza* and 7 related species, most genes appeared to be under purifying selection, while a few showed signs of positive selection, with slight variations among different species. Notably, the Ka/Ks values for *ccmB*, *mttB* and *nad6* genes in most species were greater than 1, and the *ccmB* gene in *S. sarcorrhiza* had the highest value, suggesting it was under the least selection pressure and might be a vital gene for life processes.

Codons are the fundamental units responsible for accurately identifying and transmitting genetic information [65]. Codon usage bias refers to the uneven distribution of synonymous codons that encode the same amino acid. This phenomenon is widespread among natural organisms and is influenced by various factors, including natural selection and mutation pressure [66]. By examining codon usage preferences, we can uncover the evolutionary patterns of genes and predict regulatory mechanisms during gene expression [67]. ATG serves as a common start codon in all plants, although a small number of PCGs in the mitogenome use ACG or CTG as their start codon [68]. For instance, the *nad4L* gene in the *S. sarcorrhiza* mitogenome initiates with ACG. Similar findings have been reported in other plants, such as *Rubus ideus* [55], *Ilex rotunda* [69] and *Lycopodium japonicum* [70]. This phenomenon may be attributed to RNA editing events. The RSCU value for the encoded amino acid was greater than 1, indicating that A/T nucleotide were significantly enriched at the third codon position of the *S. sarcorrhiza* gene sequence. The usage frequency of NNA and NNT codons was similar to that observed in many other species, including *Rubus suavissimus* [71], *Punica granatum* [72], and *Angelica sinensis* [63]. The pronounced bias towards A/T nucleosides at the third codon position may be a shared characteristic among the mitogenomes of various plants. Codon usage patterns reveal the non-random preference for synonymous codons by various organisms or genes, influencing mRNA stability, translation efficiency, and protein expression levels [73, 74]. In plant mitochondria, unique evolutionary pressures and genomic characteristics, such as high AT content and RNA editing, make codon usage patterns particularly significant for gene regulation. The first impact to consider is that of codon usage on translation efficiency. High-frequency codons typically correspond to abundant tRNAs in cells, enabling ribosomes to decode swiftly and minimize translation pauses, for instance, CCA (Pro), UUU (Phe), and others in plant mitochondria. The abundance of tRNAs can cause ribosomes to hesitate, potentially affecting the expression levels of protein folding box genes. Furthermore, codon usage aligns with that of highly expressed genes, which are generally more efficient in translation [75], such as the respiratory chain complex genes *cox2*, *cob*, and others. Conversely, some regulatory genes may be constrained by translation efficiency and thus regulate expression levels.

A large number of repetitive sequences are often found in the mitochondria of higher plants, including simple sequence repeats, tandem repeats and dispersed repeats [69–71]. These sequences play a crucial role in evolution. Among them, irreversible recombination mediated by short repeats with low recombination activity plays an important role, while reversible recombination mediated by long repeats with high recombination activity leads to the continuous expansion and complexity of the genome [76]. Compared to seven related species, the proportion of repeated sequences in *S. sarcorrhiza* was notably higher. Specifically, in the *S. sarcorrhiza* mitogenome, repetitive sequences made up 17.97%, which was comparable to the proportion in *Plantago ovata* (19.30%), while in other plants, this figure ranges from 2.39% to 8.49%. Additionally, extensive genetic material exchanges between organelle genomes can be observed in angiosperms, including sequence fragments transferred from plastids to mitochondria (MTPTs) and gene fragments transferred from nuclei to mitochondria [77]. MTPTs typically constitute 1-12% of the mitogenome. In *S. sarcorrhiza*, 34,978 bp MTPTs were found, which account for 5.67% of the mitogenome size, classifying them as medium-sized. Therefore, it was further proved that the size of the *S. sarcorrhiza* mitogenome was mainly affected by repetitive sequences and migrating sequences. Compared to previous studies, we observed variations in migrating fragments among different species. Annotations of MTPTs in *S. sarcorrhiza* revealed that a large number of fragments originated from plastids, providing the mitogenome with rich unnatural sequences. The complete gene may remain functional within the mitogenome. However, migrated fragments often experience varying degrees of sequence loss, and the transfer of sequences to mitochondria can also lead to the formation of pseudogenes [55]. In this study, we detected 7 complete tRNA genes within MTPTs, which represented 36.8% of mitochondrial tRNA genes. Compared to intact PCGs (14.2%), tRNA genes were more conserved in the mitogenome, indicating that they might play an indispensable role in the mitogenome. In general, sequence transfer plays a significant role in plant evolution, impacting not only the size of the mitogenome, but also increasing its genetic diversity.

RNA editing events, which involve post-transcriptional modifications of RNA sequences through substitutions, deletions or insertions, significantly contribute to transcriptome diversity and are essential steps in plant mitochondrial gene expression [78, 79]. Current research indicates that substitution is the most frequent type of editing, with the primary form being C-to-U [70, 80]. Previous studies have found that there are approximately 491 RNA editing sites in 34 PCGs of *Oryza sativa* [81], approximately 421 RNA editing sites in 35 PCGs of *Acer truncatum* [82], approximately 283 RNA editing sites in *Spodiopogon sagittifolius* [83], around 543 RNA editing sites in *Ilex metabaptista* [84], and roughly 401 RNA editing sites in *E. longipes* [85]. In the mitogenome of *S. sarcorrhiza*, 394 RNA editing sites were identified. We have observed that the number of RNA editing sites varies significantly between different species, while the number tends to be relatively conservative among closely related species. The number of RNA editing sites for different genes can vary greatly, even among closely related species. For instance, the *ccmB* gene of *E. longipes* has the most editing sites (36), whereas *nad4L* in *S. sarcorrhiza* has the highest number of RNA editing sites (35). A similar phenomenon has been noted in *R. suavissimus* and *C. camphora* [58, 71]. Such editing may affect the stability of the mitochondrial respiratory chain complex enzyme. Collinearity analysis is crucial to elucidating the evolutionary relationships among species [86]. Additionally, RNA editing can cause alterations in protein function [87]. For instance, editing within the coding region may result in non-synonymous mutations that modify protein activity; it can also disrupt enzyme active sites or protein interaction interfaces; furthermore, editing frequently introduces premature stop codons, leading to a loss of function. In the mitogenome of *S. sarcorrhiza*, RNA editing primarily occurs at the first and second codons, and the start codon of *nad4L* is altered to ACG as a result of RNA editing, a phenomenon similar to that observed in most plants [82, 85]. We conducted the collinearity tests between *S. sarcorrhiza* and six related species, respectively. The results indicated that *S. sarcorrhiza* and plants of the same family shared more homogeneous fragments, but the orders of the mitogenome sequences were extremely non-conservative, suggesting that they had experienced frequent genome recombination over a long evolutionary period. Previous studies have found that gene rearrangement is a driving factor for the remodeling of the Rosaceae mitogenome [88]. We had also observed similar results in the Lamiales mitogenome, which may be a potential driving force for the evolution of mitogenome.

The mitogenome is proof of the unique evolutionary trajectory of angiosperms [89]. The content, structure and genetic arrangement of plastomes are important for exploring the evolutionary relationships among plants [90]. In most terrestrial plants, the plastid genome size remains relatively constant, and gene losses are infrequent during organism evolution [91]. Mitochondrial genomes, on the other hand, are relatively complex and highly variable in size, yet their PCGs exhibit lower substitution rates and higher homogeneity [92]. These characteristics make the mitogenome more adept at uncovering older and deeper phylogenetic relationships within the tree of life [93]. In certain plants, discrepancies in topological structures between phylogenetic trees arise due to the distinct evolutionary paths of plastid and mitochondrial genomes [69, 94]. This study explored the phylogenetic placement of *S. sarcorrhiza* from both mitochondrial and plastids. The results showed that the phylogenetic trees constructed from plastids perspectives. The findings indicated that phylogenetic trees constructed from both plastids and mitochondria supported the classification of *S. sarcorrhiza* within the family Acanthaceae, with the plastid genome further indicating its genus as *Strobilanthes*, forming a sister group with *S. tonkinensis*, *S. dalzieli*, and *S. biocullata*, rather than *Championella*. Intriguingly, despite the high consistency in the phylogenetic position of *S. sarcorrhiza* and a very high support rate, the trees revealed minor inconsistencies in the overall phylogenetic relationships of angiosperms. For instance, the phylogenetic placement of *Vitis vinifera* was closely related to Aquifoliales in the plastid genome but closely related to Saxifragales in the mitogenome. Overall, the phylogenetic relationships inferred from mitochondrial data align more closely with the APG IV classification system. These inconsistencies might be attributed to the relatively independent genetic system of mitochondria, or they could be a result of species hybridization and ILS [95]. More accurate guesses require a more comprehensive sampling strategy and more detailed evolutionary analysis.

## Conclusions

This study assembled and annotated the mitogenome of *S. sarcorrhiza* using HiFi reads, and examined the detailed characteristics of the genome, such as GC content, codon usage preference, repetitive sequences, RNA editing events, phylogenetic relationships, sequence migration, and rearrangement. The mitogenome of *S. sarcorrhiza* featured a linear structure spanning 617,134 bp, comprising 35 PCGs, 19 tRNAs, and 3 rRNA genes, with a GC content of 42.56%. A comprehensive phylogenetic analysis indicated that it belongs to the genus *Strobilanthes*, not *Championella*. This study offers a crucial foundation for future molecular breeding, germplasm resource innovation, and phylogeny research on *S. sarcorrhiza*.

## Electronic supplementary material

Below is the link to the electronic supplementary material.

Supplementary Material 1

## Data Availability

The accession numbers of mitochondrial and plastid genomes of Strobilanthes sarcorrhiza in Gene Bank are PQ601285 and PQ631129, respectively.
